# Structure-Property Relationships in Hybrid Cellulose Nanofibrils/Nafion-Based Ionic Polymer-Metal Composites

**DOI:** 10.3390/ma12081269

**Published:** 2019-04-18

**Authors:** Colin Noonan, Mehdi Tajvidi, Ali H. Tayeb, Mohsen Shahinpoor, Seyed Ehsan Tabatabaie

**Affiliations:** 1 School of Mechanical Engineering, Gonzaga University, Spokane, WA 99258, USA; cnoonan2@zagmail.gonzaga.edu; 2 School of Forest Resources, University of Maine, 5755 Nutting Hall, Orono, ME 04469, USA; ali.tayeb@maine.edu; 3 Advanced Structures and Composites Center, University of Maine, 35 Flagstaff Road, Orono, ME 04469, USA; 4 Biomedical Engineering and Advanced Robotics Labs, Department of Mechanical Engineering, University of Maine, Orono, ME 04469, USA; shah@maine.edu (M.S.); seyed.tabatabaie@maine.edu (S.E.T.)

**Keywords:** cellulose nanofibrils, ionic polymer-metal composites, Nafion^®^, physical and mechanical properties

## Abstract

Herein, we report the production of ionic polymer-metal composites (IPMCs) hybridized with cellulose nanofibrils (CNF) as a partial substitute for Nafion^®^. The aim is not only to reduce the production cost and enhance respective mechanical/thermal properties but also to bestow a considerable degree of biodegradability to such products. Formulations with different CNF/Nafion^®^ ratios were produced in a thin-film casting process. Crack-free films were air-dried and plated by platinum (Pt) through an oxidation-reduction reaction. The produced hybrids were analyzed in terms of thermal stability, mechanical and morphological aspects to examine their performance compared to the Nafion-based IPMC prior to plating process. Results indicated that films with higher CNF loadings had improved tensile strengths and elastic moduli but reduced ductility. Thermogravimetric analysis (TGA) showed that the incorporation of CNF to the matrix reduced its thermal stability almost linearly, however, the onset of decomposition point remained above 120 °C, which was far above the temperature the composite membrane is expected to be exposed to. The addition of a cross-linking agent to the formulations helped with maintaining the integrity of the membranes during the plating process, thereby improving surface conductivity. The focus of the current study was on the physical and morphological properties of the films, and the presented data advocate the potential utilization of CNF as a nontoxic and sustainable bio-polymer for blending with perfluorosulfonic acid-based co-polymers, such as Nafion^®^, to be used in electroactive membranes.

## 1. Introduction

Ionic polymer-metal composites (IPMCs) are synthetic smart materials consisting of a proton exchange polymer fixed between metal electrodes on both sides. Due to the electroresponsive behavior; they can change shape or volume once placed under an electrical field [1,2,3]. Researchers have suggested IPMCs as sensors [4], energy harvesters [5,6], actuators, soft robotics and artificial muscles [1,2,3,7,8,9]. What makes IPMCs valuable is their unique properties, such as low density (~2 g/cm^3^), small drive voltage (0.5–7.0 V), high potential displacements (>10%) [10], low power consumption (mW-range) and the ability to be used on micro or macro scales [11,12]. Most commercial IPMCs use Nafion^®^ as their membrane (170–200 µm thick), and electroless plated platinum as electrodes (10–15 µm thick) [11]. Nafion^®^ consists of a perfluorosulfonic acid polymer with ionic side sulfonates groups. While the perfluorinated backbone has high hydrophobicity, the sulfonic acids are hydrophilic. In the presence of water, the hydrophobic backbone extends away and forms spherical clusters with sulfonic acid domains on the water surface [13,14]. Anions are not associated with the sulfonic groups, but cations can electrostatically interact with the anionic sulfonic functionalities and move/transport freely along these water channels. Under an incident electric field (via the plated metal surfaces that act as electrodes), cations migrate to the anode and pull the polar water towards the anode, creating a two-layer boundary inside the membrane. Thus, as one half will be full of cations and water molecules, the other half will be left bare. By inference, the inflation of the cation layer gives rise to an osmotic pressure that can create bending forces in the Nafion^®^ matrix [1,2,15]. Despite its acceptable performance, Nafion^®^ is very expensive, (with the average price of $600–1200 for 1 m^2^ membrane) [16], is not environmentally friendly, and requires harsh manufacturing techniques [17,18]. Many studies have been conducted to improve the performance of ionic polymers and employ them in various areas. Hong et al. evaluated the effect of metal components morphology on the electrochemical features of IPMCs and realized that the optimized morphology can enhance the IPMC sensitivity by 3–4 folds [19]. In another work, a nanoindentation test was carried out to examine the effect of temperature (due to its limiting factor in certain applications) on the elastic modulus and hardness of a proton exchange mat. The attained data showed that increasing the temperature can impart a non-monotonic increase in both features [20]. 

Lately, in response to the quest for a more affordable alternative with greener nature, cellulose nanomaterials are suggested by several authors. Cellulose nanofibrils (CNFs), a class of nanocelluloses with natural polymeric fibers, are relatively inexpensive, nontoxic, and biodegradable [21,22]. They are normally produced via mechanical grinding process in which stacked nanofibrils in the bulk cellulose are liberated to yield cellulose nanofibrils. The resulting nanomaterial can be formed into a film via a facile solution casting or filtration process at a reasonable price. Furthermore, CNFs possess fascinating features, such as large surface area, excellent tensile properties and reinforcing effect [23], low density, and unique rheology [24,25].

The presence of ample hydroxyl groups on the surface has granted cellulose a high water uptake capacity, tunable chemistry, and ability to establish hydrogen bonds within polymeric structures [22,26]. The water-loving nature of these nanoscopic entities can provide possible aid to the highly hydrophilic sulfonic groups to form ionic clusters for ions and water transport. In the literature, nanocelluloses have been suggested for applications in photonic films [27], polymer electrolyte membranes [28], fuel cells [29], and polymer-metal actuators [30]. Besides this, other types of nanocelluloses have been utilized for related systems. For instance, films that were produced from bacterial cellulose (BC) and Nafion^®^ (through solution casting method) displayed positive ionic conductivity and maintained acceptable thermostability under 100 °C, both of which are critical for proton-exchange membrane fuel cells (PEMFCs) and/or direct methanol fuel cells (DMFCs) [29]. In a pertinent study by Gadim et al., a crack-free hybrid membrane was developed from bacterial cellulose and Nafion^®^. The resulting matrix showed high mechanical properties and an in-plane protonic conductivity of 0.14 S·cm^−1^ [28]. Moreover, cellulose nanowhiskers (known as cellulose nanocrystals) have been successfully included to Nafion^®^ membrane to maximize the fuel cell performance. The analysis on electrochemical impedance showed addition of 5 wt.% nanowhiskers can positively affect the proton conductivity at a temperature above 100 °C. They also noticed that the presence of cellulose gives rise to lower methanol permeability [20]. However, the use of CNF with Nafion^®^ to manufacture IPMCs, which is the focus of this study, has not been reported yet.

Over the last 30 years, our society has slowly made the transformation from paper to digital and has left the paper and wood industry with a surplus of raw wood. This has created an abundance of wood material that can potentially be used to produce CNF. Moreover, finding novel applications for CNF can help revitalize the declining pulp and paper industry. In the current study, upon optimization, we are proposing a new ionic polymer-metal composite with a 50:50 ratio of CNF and Nafion in the presence of 2% crosslinking agents, namely polyamidoamine-epichlorohydrin and Acrodur. The cross-linkers were adopted based on our previous study in which their inclusion could significantly boost the robustness in CNF-based films [31]. The membranes were platinum plated in an electroless process, and the attained CNF-containing IPMCs were analyzed in terms of thermostability, tensile strength, elastic modulus, ion conductivity, and morphology.

## 2. Materials and Methods

**Figure 1:**
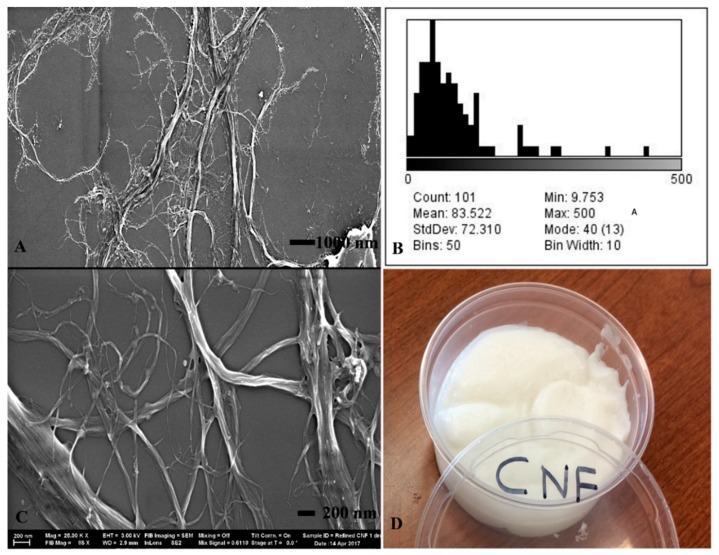
SEM of pure cellulose nanofibrils (CNF) used in the formulation of hybrid membranes: At 5K magnification (**A**) and 25K magnification (**C**). Fibril width distribution histogram is presented in (**B**), physical appearance of a 3% by weight solids CNF suspension is seen in (**D**).

CNF (3 wt.% solids) was produced at the University of Maine Process Development Center (Orono, ME, USA) and used as the substrate for hybrid membranes. Figure 1 presents the scanning electron microscopy, fibril width distribution, and physical appearance of the CNF material. The CNF had an average sub-micron fibril width of 83 nm, with a wide range of distribution (CV = 87%). The length scale of the fibrils was in micron range, giving the material a very high aspect ratio. The CNF was produced through mechanical refining of bleached Kraft softwood pulp with a reported zeta potential of −48 to −5 mV. A 5% solution of Nafion^®^ (1100 equivalent weight) in a 50:50 mix of water and 1-propanol was purchased from DuPont (Wilmington, DE, USA) as the base membrane for IPMCs. Tetraamine platinum (II) chloride Pt(NH_3_) 4HCl, sodium borohydride (NaBH4), hydroxylamine hydrochloride, and ammonium hydroxide were applied to electroplate the outer surface of the IPMCs and were purchased from Sigma-Aldrich (St. Louis, MO, USA). Polyamidoamine-epichlorohydrin (also known as Kymene) and Acrodur are crosslinking additives used to strengthen the membranes. Poly(ethylene oxide) was used to enhance the thermostability of CNF-Nafion^®^ membranes. Acrodur was supplied by BASF Chemical Company (Ludwigshafen, Germany) and polyamidoamine-epichlorohydrin was supplied from Solenis (Wilmington, DE, USA). Carboxylate styrene-butadiene latex (GENFLO 5086) was used as an additive and obtained from OMNOVA Solutions (Fairlawn, OH, USA).

### 2.1. Production of Hybrid Membranes

CNF (3 wt.%) was diluted to 1.5% solids content and sonified for eight minutes using a Branson Sonifier 450 VWR Scientific (Danbury, CT, USA) for better dispersion of nanoparticles in the suspension. Ratios of 80:20, 65:35, 50:50, 35:65, and 20:80 (by weight) CNF to Nafion were combined using a THINKY planetary centrifugal mixer (Tokyo, Japan) for 10 minutes to provide uniform mixing and remove air bubbles. The solution-casted membranes were air-dried under a laboratory hood for about 20 h and carefully detached from the petri dishes before they began to bend. 

### 2.2. Thermogravimetric Analysis (TGA)

The thermal stability of CNF-Nafion^®^ membranes was determined using a TA Instruments Q500 thermogravimetric analyzer (New Castle, PA, USA). Thermographs were analyzed using the TA Instruments Universal Analysis 2000 software. Two samples from each group were evaluated at a 10 °C/min ramp, over a temperature range of 20–600 °C, in nitrogen atmosphere. Sample weights varied from 2 to 8 mg.

### 2.3. Scanning Electron Microscopy

The morphologies (surface and fibril spacing, thickness) of CNF, Nafion^®^, and different ratios of CNF-Nafion hybrids were examined by a Hitachi TM3000 Tabletop Microscope (Tokyo, Japan). The Hitachi SEM did not require sputter coating, and samples were examined at various magnifications.

### 2.4. Tensile Testing

Mechanical properties of the membranes were determined using an Instron 5942 equipped with a 500N load cell (Instron, Norwood, MA, USA) according to ASTM D-882 with modifications. Membranes were cut from circular films into lengths of 30–40 mm and widths of 10 mm. The gauge length was 22 mm. Thicknesses ranged from 40 to 80 microns. Tests were run at 2.00 mm/min under 23 °C and 50% relative humidity. At least five specimens were tested for all the formulations except for the pure Nafion films that had two replications due to the difficulty in solution-casting the pure membranes.

### 2.5. Sputter Coating

As an alternative plating method, a Cressington 108AutoSEM sputter coater (Cressington Scientific Instruments Limited, Watford, UK) was used to sputter coat membranes to lower surface resistance on the electrodes. The utilized metal was a composite of gold and palladium. Each run deposited around 35 nm of metal in 90 s at 40 mA, with a working distance of 50 mm (nominal) under 0.08 mBar (argon) pressure. Two runs were applied on each side of the membranes. Films were tested for sheet electrical resistance using a Micronta (22-204U) multimeter (Fort Worth, TX, USA).

### 2.6. Statistical Analysis

A one-way analysis of variance (ANOVA) followed by a Duncan’s multiple range test (DMRT) were performed on Young’s modulus, tensile strength, and tensile-strain data. In all figures presented in this paper, data points with common letters are not statistically significant at a 95% confidence level.

## 3. Results

### 3.1. Morphology and Microstructure 

**Figure 2:**
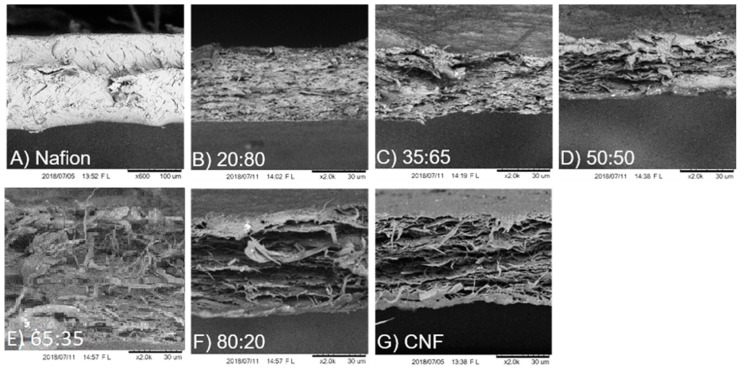
SEM cross-sectional images of Nafion^®^ at 600× magnification (**A**), Pure CNF (**G**), and different ratios of CNF to Nafion^®^ membranes after rupture in tensile analysis: 20:80 (**B**), 35:65 (**C**), 50:50 (**D**), 65:35 (**E**), 80:20 (**F**) at 2K magnification. The average thickness was 50 µm.

The cross section of produced films from pure Nafion^®^, pure CNF and CNF-Nafion^®^ hybrids with different ratios (20:80, 35:65, 50:50, 65:35, and 80:20 CNF to Nafion^®^) were investigated by SEM, and the results are displayed in Figure 2. Fractured surfaces of specimens (from tensile analysis) were imaged after failure to examine differences in morphology and ductility. As shown in Figure 2A, pure Nafion^®^ was condensed and exhibited lower porosity and fewer cracks relative to CNF. The observed necking (data not shown) after prolonged strain may also suggest its ductility. Figure 2G shows pure CNF films with higher porosity, consisting of many horizontally stacked sheets. CNF films did not undergo as large of strains as Nafion^®^, but under SEM, the CNF breaking point shows rope-like cellulose fibrils that extend perpendicular off the cross section of the break. These rope-like cellulose fibrils are groups of microfiber clusters (1–2 µm thick) that extend out 15–20 µm from the ruptured area. In the produced composites, CNF maintained its unique sheet-like structure, while Nafion^®^ seemed to blend homogeneously. Studies have shown that the microstructure of Nafion^®^ can be disrupted once incorporated into composites, and it tends to gather on the high surface area particles. This can explain why Nafion^®^ took the shape of CNF fibers, whose nanoscale fibrils enable it to have high surface area [32]. At higher ratios of CNF, the rope-like cellulose fibers were more apparent. At high CNF loads, more layers were formed, but at lower percentages films became denser and less porous.

### 3.2. Mechanical Properties

**Figure 3:**
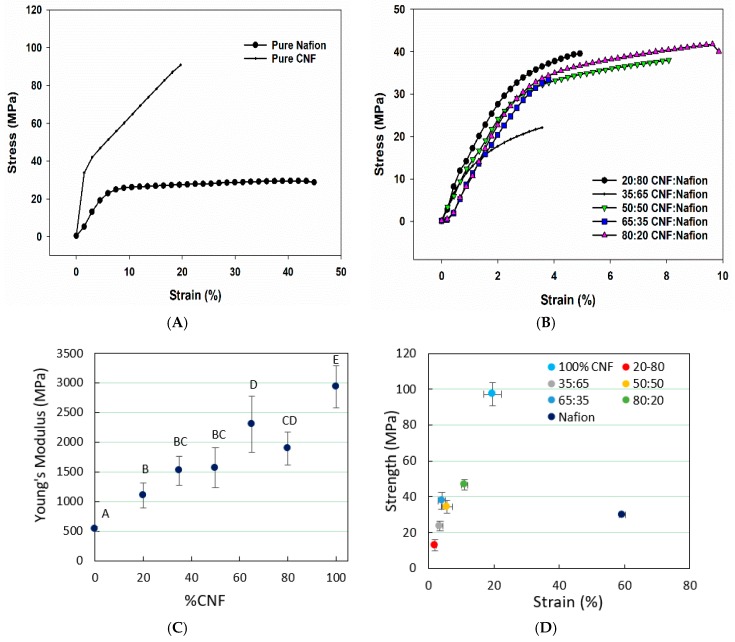
Graphs showing the typical stress-strain curves of pure materials and the hybrids thereof (**A** and **B**, respectively), tensile data obtained from CNF-Nafion^®^ membranes with different CNF contents: (**C**) Young’s modulus and (**D**) tensile strength-strain relationship. Data points with common letters are not statistically significant at 95% confidence level, as determined by statistical analysis. In (**D**), all the ratios follow the same CNF/Nafion^®^ order.

Typical stress-strain curves of pure Nafion^®^ and pure CNF are presented in Figure 3A, and a comparison of stress-train curves for the hybrid membranes is given in Figure 3B. Figure 3C shows the Young’s modulus over percentage of CNF in Nafion^®^-CNF membranes. Obtained results showed that higher CNF ratios increased Young’s modulus, which agrees with our SEM observation regarding CNF’s sheet-like structure and stiffer membranes. As shown in Figure 3D, pure Nafion^®^ has the highest strain at break (59.4%), followed by pure CNF at 19.6% strain. In general, samples with higher CNF content have higher strain, but the strain values are dramatically lower than pure Nafion^®^. It was also revealed that addition of CNF gives rise to a higher tensile strength (Figure 3D). Pure Nafion^®^ had a tensile strength of 29.9 MPa, which seems to fall in between membranes with 35:65 and 50:50 CNF/Nafion^®^ ratios. Relatively, CNF and Nafion^®^ are both tough materials, however, when they are formed into a composite, overall ductility may decrease. 

### 3.3. Thermal Stability

**Figure 4:**
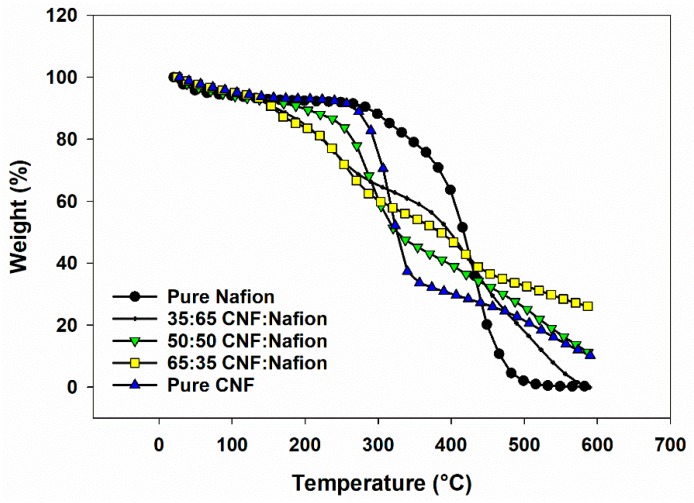
TG graph of membranes of Nafion^®^, CNF and hybrids of Nafion-CNF with different ratios in nitrogen atmosphere.

**Figure 5:**
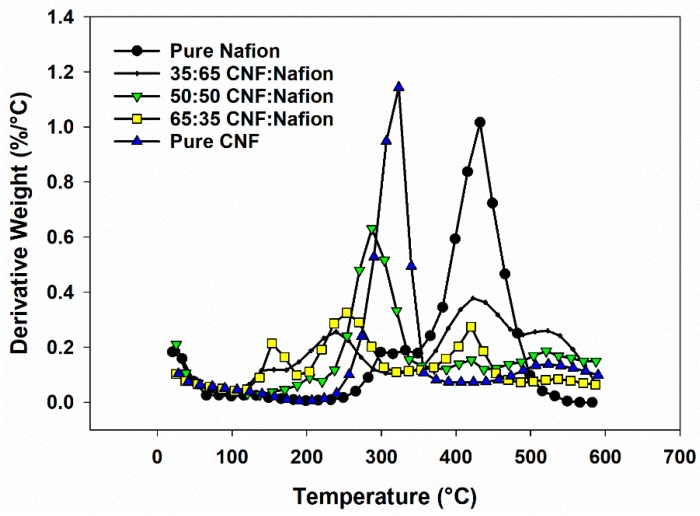
Derivative thermogravimetric (DTG) profiles of the same membranes: Nafion^®^, CNF and hybrids of Nafion^®^ in nitrogen atmosphere.

Figure 4 and Figure 5 summarize the thermogravimetric data for CNF-Nafion^®^ membranes. Seven samples ranging from pure Nafion^®^ to pure CNF were investigated. For pure CNF, the most weight loss occurred between the temperatures of 200–375 °C and was ascribed to the thermal cleavage of glyosidic linkages of cellulose [33,34]. The average onset temperature of thermal degradation for pure CNF was 271 °C, and the average temperature where maximum weight loss occurred was 322 °C, which is in line with reported studies in literature [33,35]. Pure Nafion^®^ began to deteriorate at 391 °C and reached its max peak temperature of weight loss at 436 °C, in agreement with the literature [36]. A slight weight change between 260–360 °C can be the breakage of sulfonic groups from Nafion^®^ side chains, and the mass loss in 375–500 °C was caused by degradation of the polymer backbone [37]. For all hybrids, there were four main weight loss stages: (1) a 7–12% loss at 120 °C related to the vaporization of water, (2) loss at 120–200 °C as a result of dehydration reaction between the sulfoacid groups in Nafion and the hydroxyl groups of CNF [29], (3) weight reduction in 200–350 °C due to CNF degradation and (4) the weight loss associated to Nafion decomposition. With higher Nafion^®^ content, more degradation occurred in the second and fourth stages, and less in the third stage. Average moisture content was increased 2–8% as CNF and Nafion^®^ were blended—the highest moisture content in the evaluated formulations belonged to the 65:35 CNF/Nafion^®^ formulation. Overall, all composites showed onset degradation points above 100 °C, which still enables them to be used as artificial muscles and low temperature sensors and actuators.

### 3.4. Sputter Coating

It is reported that sputter coating of gold on top of platinum plated membranes can lower the surface resistance and offer a thin layer of very conductive material on the surface [38]. One sample of pure Nafion and one of the 65:35 Nafion^®^ to CNF were sputter coated on both sides (twice) to the thickness of 70 nm. Both samples showed varied electrical resistance from 50 to 800 Ω. An optimum electrode thickness was not reached using this technique, and, therefore, no further sputter coating was carried out for IPMCs.

### 3.5. Chemical Plating by Oxidation-Reduction

An oxidation-reduction reaction (Redox) was used for the platinum plating process of the composite films. The process was adopted from earlier publications [1,2,39] with few minor adjustments, owing to the incorporation of CNF. The plating process for Nafion^®^ usually consists of four steps: (1) roughening the surface of the material with sandpaper to increase the surface area where platinum salt penetrations occur along with immersing in acid (HCl) for protonation, (2) ion exchange of tetra-amine platinum chloride hydrate (in aqueous form) with hydrogen ions, (3) an initial platinum compositing process using sodium borohydride to reduce platinum cations to the metallic state, and (4) a final surface electroding process via the same reduction process of platinum to reduce the surface resistance of the electrode. The final step is usually repeated up to four times to reduce the electrical resistance [15,38].

**Figure 6:**
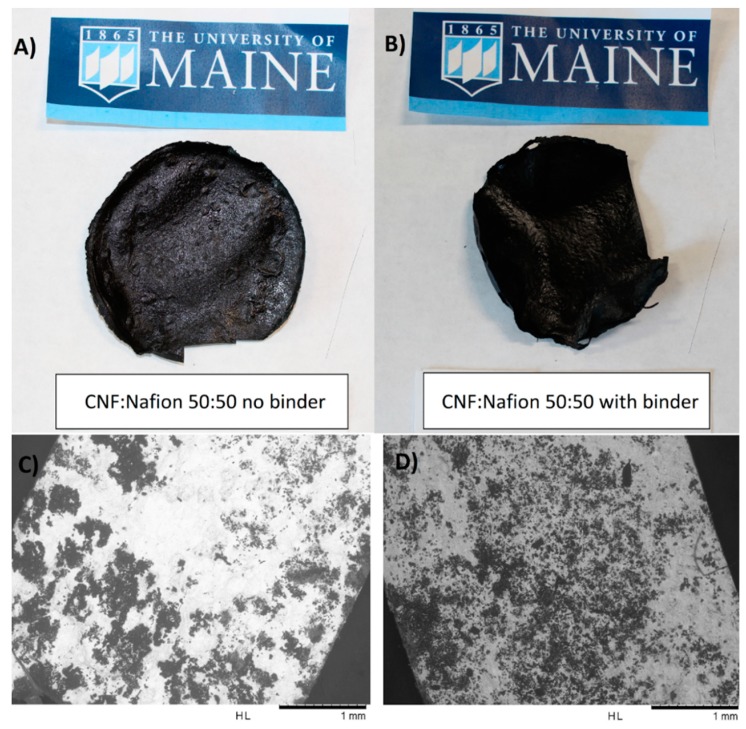
Photographs of selected CNF-Nafion^®^ ionic polymer-metal composite (IPMC) membranes with/without binder at 50:50 CNF/Nafion^®^ ratio, as made by electroless chemical plating of platinum (**A**,**B**), and example SEMs of acceptable (**C**) versus unacceptable (**D**) plated sides of a 50:50 CNF/Nafion^®^ sample, showing differences in the quality of plating on different sides.

Because of the CNF structure, adjustments were made to the procedure to optimize mechanical and ionic properties. The solution-casting method used for the CNF-Nafion^®^ films resulted in one rough side due to the exposure to air. The other side attaching to the flat petri dish was quite smooth. We examined both sides to see which one would be plated more effectively. Grey platinum layers were visible on the rough side, indicating a successful final surface electroding, while the smooth side remained black, meaning additional platinum was not well plated. SEMs of these two plated sides are presented in Figure 6C,D, where better plating was observed on the rougher surface as compared with the smoother one. On a 50:50 CNF/Nafion^®^ film, the rough grey side exhibited drastically lower surface resistance across the whole film, averaging around 14 Ω with a standard deviation of 18 Ω, while the smooth black side had a high resistance, above the limit of the measuring device. The addition of 2% cross-linker (polyamidoamine-epichlorohydrin), shown in Figure 6 on the right side, assisted the plating process via its positive role in better holding the CNF-Nafion^®^ membrane together during the ion exchange reaction. These films had an average resistance of almost zero Ω on the good side versus a resistance of 2 Ω on the bad side, with a standard deviation of 2 Ω. 

In the plating of pure Nafion^®^, after a mild sanding films were immersed in hydrochloric acid to enhance their ionic conductivity and dope the membrane with protons [40]. It is reported that doping CNF and Nafion^®^ with high concentrations of phosphoric or hydrochloric acid at room temperature can result in a greater ionic conductivity and less resistant electrodes [41]. Despite the cellulose recalcitrant structure, exposure to the aforesaid mild acidic condition led to a partial hydrolysis. For that reason, in the case of CNF-Nafion^®^ membranes with no cross-linkers, they became brittle and disintegrated, especially in membranes containing less than 65% Nafion^®^. Thus, in our study, the presence of a cross-linker was essential to maintain the membranes’ integrity.

### 3.6. Ionic Conductivity 

Ion conductivity was tested using a signal generator and measured by the displacement of the IPMC. Membranes of 35:65 and 50:50 CNF-Nafion with 2% Polyamidoamine-epichlorohydrin binder produced small displacements of 1–2 mm. Results were not as good as pure Nafion^®^ IPMCs that displayed an approximate displacement of 10–20 mm, but even the small movement in the hybrids pointed to creation of ion-carrying channels that could allow a limited pressure gradient. Pieces of the membrane on the outside rim showed greater movement than the inner parts. It appeared that the pieces on the outer rim were plated better and exhibited lower and constant surface resistance around 1–2 Ω. The change in IPMC displacement did not alter with the amount of Nafion^®^ at the rim of the film. One possibility for lower electric response of the produced membranes can be due to the induced higher porosity. While the pure Nafion^®^ membrane had a calculated porosity of about 12%, porosity for hybrid membranes was 28%, 42%, 40%, 37%, and 34% as CNF content varied from 20% to 80%. In a follow-up effort, the abovementioned membrane was pressed at mild temperatures to increase its density and improve the films’ electric response. 

It was found elsewhere that in pure Nafion^®^ membranes, clusters of sulfonate groups can become isolated within the fluorocarbon cage, making sulfonates inaccessible to cations. This can decrease the hydrophilic boundary and make the protons channels less apparent—25% of exchange sites were inaccessible in 1100 equivalent weight Nafion^®^. The research reported that the application of CNF as a substrate can disperse the ionic clusters, allowing more sulfonate groups to be accessible for creating better ionic channels [32].

### 3.7. Incorporation of Additives 

Additives were included in some samples to improve flexibility, thermostability, and limit degradation in the electrode plating process. Carboxylate styrene-butadiene latex (GENFLO 5086) with 2% weight and Acrodur with 2% weight were added to a 50:50 CNF-Nafion film to improve flexibility. This composite membrane failed to be removed from a glass petri dish, and only half the film survived detachment from a plastic petri dish. No mechanical tests were continued for these samples. Films of 40:40:20 and 45:45:10 CNF/Nafion/Poly(ethylene oxide) were solution-casted for the potential of improving thermostability due to poly(ethylene oxide)’s ability to enhance thermostability of cellulose nanocrystals (CNC) [42]. However, thermograms (not presented here) showed no substantial increase in thermostability. As mentioned, polyamidoamine-epichlorohydrin and Acrodur were added to the 50:50 and 35:65 CNF/Nafion^®^ membranes at 2% weight. They both acted as cross-linkers for CNF and Nafion^®^ that were expected to strengthen the material and keep the membranes from disintegrating when strong acids were introduced during the plating process. By observation, it appears the addition of polyamidoamine-epichlorohydrin helped the electroplating process because more platinum precipitated on the surface of the membrane and lowered the surface resistance. More importantly, incorporation of this cross-linker enhanced the films’ integrity and prevented their disintegration during the proton-doping process.

## 4. Conclusions

In summary, various hybrid membranes of CNF and Nafion^®^ were fabricated to be used as an IPMC. Using scanning electron microscopy, each film was examined for differences in morphology, and it was revealed that specimens with higher CNF content were more porous and less dense, while films with pure Nafion^®^ displayed opposite features. Each film was analyzed for thermostability. A new weight loss period occurred between 120 °C and 200 °C because of a possible dehydration reaction between the hydroxyl groups of CNF and the sulfonic groups of Nafion^®^, as reported by others [41,43]. Films with a higher CNF content degraded between 200 and 350 °C while those with more Nafion^®^ deteriorated between 350 and 500 °C. With respect to mechanical attributes, the presence of CNF led to a higher elastic modulus, tensile strengths and stiffness, however, strain values were decreased markedly, pointing to a more fragile structure. Finally, small displacements of 35:65 and 50:50 CNF/Nafion^®^ IPMCs with 2% weight polyamidoamine-epichlorohydrin were recorded, showing promise for future improvements. Overall, the current study suggests the potential of a hybrid IPMC from Nafion^®^ and CNF with enhanced mechanical and thermal stability. The observed drawback was lower electroresponsive properties in the attained hybrids compared to the pure Nafion^®^. With optimized plating methods and control of density, the CNF-Nafion^®^ hybrid membranes could serve as artificial muscles and low-temperature sensors and actuators. High concentration of weak acids such as phosphoric and phytic acid were shown to improve bacterial cellulose ion conductivity, creating potential to improve ionic conductivity in CNF and use higher CNF content in CNF-Nafion^®^ hybrid membranes. It is unclear yet how the hydrophilicity of CNF affects the hydrophobic backbone and hydrophilic side groups. The answer can be critical in revealing whether the combination tightens, separates, or creates more channels for ions and water to pass.

## References

[1] Shahinpoor M., Kim K.J. (2000). The effect of surface-electrode resistance on the performance of ionic polymer-metal composite (IPMC) artificial muscles. Smart Mater. Struct..

[2] Shahinpoor M., Kim K.J. (2001). Ionic polymer-metal composites: I. Fundamentals. Smart Mater. Struct..

[3] Shahinpoor M., Kim K.J. (2005). Ionic polymer–metal composites: IV. Industrial and medical applications. Smart Mater. Struct..

[4] Shen Q., Wang T., Kim K.J. (2015). A biomimetic underwater vehicle actuated by waves with ionic polymer–metal composite soft sensors. Bioinspir. Biomim..

[5] Vinh N.D., Kim H.-M. (2017). Ocean-based electricity generating system utilizing the electrochemical conversion of wave energy by ionic polymer-metal composites. Electrochem. Commun..

[6] Aureli M., Prince C., Porfiri M., Peterson S.D. (2010). Energy harvesting from base excitation of ionic polymer metal composites in fluid environments. Smart Mater. Struct..

[7] Yong Jung S., Young Ko S., Park J.-O., Park S. (2015). Enhanced ionic polymer metal composite actuator with porous nafion membrane using zinc oxide particulate leaching method. Smart Mater. Struct..

[8] Ru J., Wang Y., Chang L., Chen H., Li D. (2016). Preparation and characterization of water-soluble carbon nanotube reinforced Nafion membranes and so-based ionic polymer metal composite actuators. Smart Mater. Struct..

[9] Kaal W., Herold S. (2011). Electroactive Polymer Actuators in Dynamic Applications. IEEE/ASME Trans. Mechatron..

[10] Leary S.P., Bar-Cohen Y., Bar-Cohen Y. (1999). Electrical impedence of ionic polymeric metal composites. Smart Structures and Materials.

[11] Bar-Cohen Y. (2004). Electroactive Polymer (EAP) Actuators as Artificial Muscles: Reality, Potential, and Challenges.

[12] Asaka K., Oguro K., Nishimura Y., Mizuhata M., Takenaka H. (1995). Bending of Polyelectrolyte Membrane–Platinum Composites by Electric Stimuli I. Response Characteristics to Various Waveforms. Polym. J..

[13] Napoli L., Lavorante M., Franco J., Sanguinetti A., Fasoli H. (2013). Effects on Nafion^®^ 117 Membrane using Different Strong Acids in Various Concentrations. J. New Mater. Electrochem. Syst..

[14] Kreuer K.D. (2001). On the development of proton conducting polymer membranes for hydrogen and methanol fuel cells. J. Memb. Sci..

[15] Zengin T. (2012). Fabrication, Characterization and Modeling of Electroactive Polymer based Smart Structures for a Biological-like Artificial Muscle. Ph.D. Thesis.

[16] Neburchilov V., Martin J., Wang H., Zhang J. (2007). A review of polymer electrolyte membranes for direct methanol fuel cells. J. Power Sources.

[17] Kraytsberg A., Ein-Eli Y. (2014). Review of Advanced Materials for Proton Exchange Membrane Fuel Cells. Energy Fuels.

[18] Hwang T., Palmre V., Nam J., Lee D.-C., Kim K.J. (2015). A new ionic polymer–metal composite based on Nafion/poly(vinyl alcohol- *co* -ethylene) blends. Smart Mater. Struct..

[19] Hong W., Almomani A., Montazami R. (2017). Electrochemical and morphological studies of ionic polymer metal composites as stress sensors. Measurement.

[20] Xia R., Zhou H., Wu R., Wu W.-P., Xia R., Zhou H., Wu R., Wu W.-P. (2016). Nanoindentation Investigation of Temperature Effects on the Mechanical Properties of Nafion^®^ 117. Polymers.

[21] Azeredo H.M.C., Mattoso L.H.C., Wood D., Williams T.G., Avena-Bustillos R.J., McHugh T.H. (2009). Nanocomposite Edible Films from Mango Puree Reinforced with Cellulose Nanofibers. J. Food Sci..

[22] Moon R.J., Martini A., Nairn J., Simonsen J., Youngblood J. (2011). Cellulose nanomaterials review: Structure, properties and nanocomposites. Chem. Soc. Rev..

[23] Yadav M., Chiu F.-C. (2019). Cellulose nanocrystals reinforced κ-carrageenan based UV resistant transparent bionanocomposite films for sustainable packaging applications. Carbohydr. Polym..

[24] Hubbe M.A., Tayeb P., Joyce M., Tyagi P., Kehoe M., Dimic-Misic K., Pal L. (2017). Rheology of nanocellulose-rich aqueous suspensions: A review. BioResources.

[25] Chinga-Carrasco G., Syverud K. (2010). Computer-assisted quantification of the multi-scale structure of films made of nanofibrillated cellulose. J. Nanopart. Res..

[26] Tayeb A.H., Amini E., Ghasemi S., Tajvidi M. (2018). Cellulose Nanomaterials—Binding Properties and Applications: A Review. Molecules.

[27] Guidetti G., Atifi S., Vignolini S., Hamad W.Y. (2016). Flexible Photonic Cellulose Nanocrystal Films. Adv. Mater..

[28] Gadim T.D.O., Vilela C., Loureiro F.J.A., Silvestre A.J.D., Freire C.S.R., Figueiredo F.M.L. (2016). Nafion^®^ and nanocellulose: A partnership for greener polymer electrolyte membranes. Ind. Crops Prod..

[29] Jiang G., Zhang J., Qiao J., Jiang Y., Zarrin H., Chen Z., Hong F. (2015). Bacterial nanocellulose/Nafion composite membranes for low temperature polymer electrolyte fuel cells. J. Power Sources.

[30] Shen Q., Trabia S., Stalbaum T., Palmre V., Kim K., Oh I.-K. (2016). A multiple-shape memory polymer-metal composite actuator capable of programmable control, creating complex 3D motion of bending, twisting, and oscillation. Sci. Rep..

[31] Tayeb A.H., Tajvidi M. (2018). Sustainable Barrier System via Self-Assembly of Colloidal Montmorillonite and Cross-linking Resins on Nanocellulose Interfaces. ACS Appl. Mater. Interfaces.

[32] Chen T.-Y., Johna L. (2000). Ion Exchange Capacity of Nafion and Nafion Composites. Langmuir.

[33] Xu C., Zhu S., Xing C., Li D., Zhu N., Zhou H. (2015). Isolation and Properties of Cellulose Nanofibrils from Coconut Palm Petioles by Different Mechanical Process. PLoS ONE.

[34] Manfredi L.B., Rodríguez E.S., Wladyka-Przybylak M., Vázquez A. (2006). Thermal degradation and fire resistance of unsaturated polyester, modified acrylic resins and their composites with natural fibres. Polym. Degrad. Stab..

[35] Yildirim N., Shaler S., Yildirim N., Shaler S. (2017). A Study on Thermal and Nanomechanical Performance of Cellulose Nanomaterials (CNs). Materials.

[36] Deng Q., Wilkie C.A., Moore R.B., Mauritz K.A. (1998). TGA–FTi.r. investigation of the thermal degradation of Nafion^®^ and Nafion^®^/[silicon oxide]-based nanocomposites. Polymer.

[37] Zhang F., Zhang Z., Liu Y., Lu H., Leng J. (2013). The quintuple-shape memory effect in electrospun nanofiber membranes. Smart Mater. Struct..

[38] Lee S.G., Park H.C., Pandita S.D., Yoo Y. (2006). Performance improvement of IPMC (ionic polymer metal composites) for a flapping actuator. J. Control. Autom. Syst..

[39] Kim K.J., Shahinpoor M. (2003). Ionic polymer metal composites: II. Manufacturing techniques. Smart Mater. Struct..

[40] Kuwertz R., Kirstein C., Turek T., Kunz U. (2016). Influence of acid pretreatment on ionic conductivity of Nafion^®^ membranes. J. Memb. Sci..

[41] Jiang G., Qiao J., Hong F. (2012). Application of phosphoric acid and phytic acid-doped bacterial cellulose as novel proton-conducting membranes to PEMFC. Int. J. Hydrog. Energy.

[42] Ben Azouz K., Ramires E.C., Van den Fonteyne W., El Kissi N., Dufresne A. (2012). Simple Method for the Melt Extrusion of a Cellulose Nanocrystal Reinforced Hydrophobic Polymer. ACS Macro Lett..

[43] Roman M., Winter W.T. (2004). Effect of Sulfate Groups from Sulfuric Acid Hydrolysis on the Thermal Degradation Behavior of Bacterial Cellulose. Biomacromolecules.

